# Late-Proterozoic to Paleozoic history of the peri-Gondwana Calabria–Peloritani Terrane inferred from a review of zircon chronology

**DOI:** 10.1186/s40064-016-1839-8

**Published:** 2016-02-29

**Authors:** Annamaria Fornelli, Francesca Micheletti, Giuseppe Piccarreta

**Affiliations:** Department of Earth and Geoenvironmental Science, University of Bari, Via E. Orabona 4, 70125 Bari, Italy

**Keywords:** U–Pb zircon ages, Pre-Cambrian to Permian tectonothermal events, Detrital provenance, Calabria–Peloritani Terrane

## Abstract

U–Pb analyses of zircon from ten samples of augen gneisses, eight mafic and intermediate metaigneous rocks and six metasediments from some tectonic domains along the Calabria–Peloritani Terrane (Southern Italy) contribute to knowledge of peri-Gondwanan evolution from Late-Proterozoic to Paleozoic times. All samples were equilibrated under amphibolite to granulite facies metamorphism during the Variscan orogeny. The zircon grains of all considered samples preserve a Proterozoic memory suggestive of detrital, metamorphic and igneous origin. The available data fit a frame involving: (1) Neoproterozoic detrital input from cratonic areas of Gondwana; (2) Pan-African/Cadomian assemblage of blocks derived from East and West African Craton; (3) metamorphism and bimodal magmatism between 535 and 579 Ma, within an active margin setting; (4) rifting and opening of Ordovician basins fed by detrital input from the assembled Cadomian blocks. The Paleozoic basins evolved through sedimentation, metamorphism and magmatism during the Variscan orogeny involving Palaeozoic and pre-Paleozoic blocks. The Proterozoic zircon records decidedly decrease in the high grade metamorphic rocks affected by Variscan pervasive partial melting.

**Electronic supplementary material:**

The online version of this article (doi:10.1186/s40064-016-1839-8) contains supplementary material, which is available to authorized users.

## Introduction

Amalgamation and break up of supercontinents and superterranes (Rodinia, Gondwana, Pangea) characterize the history of the Earth between Neoproteozoic and Palaeozoic times. All geological processes known today, starting with Rodinia fragmentation and culminating with the assemblage of Pangea, have been the focus of research in recent decades (e.g. von Raumer et al. 2013, 2015 and references therein). Records of magmatism, sedimentation, metamorphism and anatexis accompanying the evolution of the superterrane Gondwana are preserved in some tectonic units of the nappe structured Calabria–Peloritani Terrane (CPT, Southern Italy) reworked by the Variscan and Alpine orogenies. This terrane, according to the most recent paleogeographic reconstructions, was one of the peri-Gondwanan blocks comprising the “Galatian superterrane” (Stampfli et al. 2011; von Raumer et al. 2013 and references therein).

A large number of geochronological data obtained using microbeam techniques (SIMS, LA-ICP-MS, SHRIMP), together with previous data collected through traditional methods (ID-Tims) are today available on different rock types exposed in this area (e.g. Schenk 1980, 1989, 1990; Senesi 1999; Trombetta et al. 2004; Micheletti et al. 2007, 2008, 2011; Langone 2008; Laurita et al. 2015; Fiannacca et al. 2008, 2013; Fornelli et al. 2011a, 2014; Williams et al. 2012). These data, together with the zircon grain growth textures revealed by SEM imaging (cathodoluminescence-CL and variable pressure secondary electron-VPSED) and the REE–U–Th distribution in the zircon domains, can contribute to: (1) estimate the age and nature of magmatic products, (2) infer the minimum sedimentation ages of the protoliths of some metasedimentary rock types, (3) determine the role of temperature and fluids or melts on the resetting or new growth of zircon and, finally, (4) establish the provenance of detrital materials. In this paper, the available data have been reappraised to provide a synthetic frame in which the above processes occurred. A synthesis of available age data in Calabria Peloritani Terrane, dispersed in many papers, could contribute to clarify the paleogeographic renconstruction of peri-Gondwanan blocks.

**Fig. 1:**
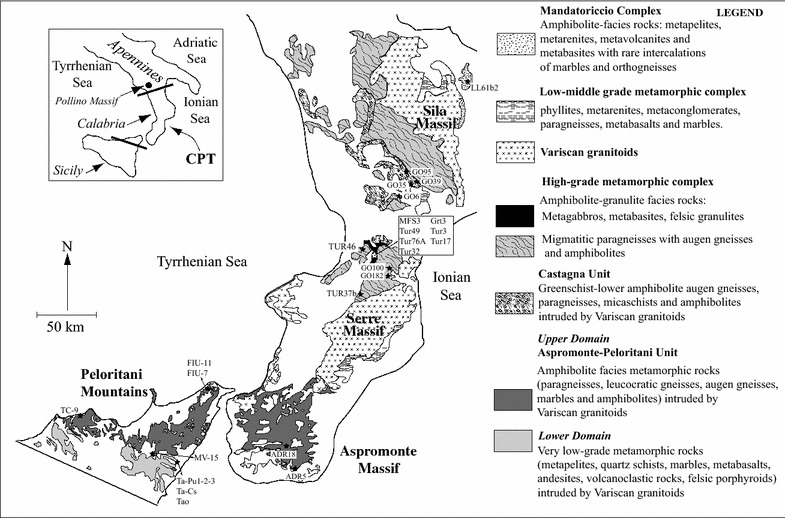
Distribution of the studied rock Units and location of the considered samples in Calabria (southern Italy)

Attention is focused on metasediments, augen gneisses and metabasites from four structural domains of the Calabria–Peloritani Terrane: Mandatoriccio complex, Castagna unit, high-grade metamorphic complex in Serre massif, Aspromonte–Peloritani unit in Aspromonte massif and Sicily (Fig. 1). One sample of garnet-biotite gneiss considered comparable to the high-grade metasediments of the Calabria deep crust, derived from a sliver within the Alpine accretionary wedge along the Calabria–Lucanian boundary (Pollino Massif insert Fig. 1), was also considered for the zircon age data (Laurita et al. 2015).

All rocks experienced Variscan metamorphism under conditions ranging from amphibolite (Castagna, Mandatoriccio and Aspromonte–Peloritani Unit) to granulite facies (high-grade metamorphic complex).

## Geological background

The Calabria–Peloritani Terrane (CPT) is an “exotic terrane” (Bonardi et al. 2001) comprising a Pre-Mesozoic basement consisting of different tectonic units affected by Variscan metamorphism and stacked during the Alpine orogenesis. It includes the crystalline massifs of Calabria (Sila, Serre and Aspromonte) and Peloritani Mountains of Sicily (Fig. 1). In addition, slivers of garnet-biotite gneisses considered as equivalent to the high-grade metasediments of the Serre massif, occur in the Alpine tectonic mélange at the Calabria–Lucania boundary (Pollino Massif insert Fig. 1).

The nappe belt includes, in northern Calabria, from the top to the bottom: (1) pre-Triassic basements with metamorphic and igneous rocks pertaining to the south-European Variscan belt; (2) fragments of Jurassic to lower Cretaceous Tethyan oceanic crust affected by Alpine HP/LT metamorphism; and (3) Mesozoic to Cenozoic sedimentary rocks at the bottom (e.g. Amodio Morelli et al. 1976; Bouillin et al. 1984). In Southern Calabria (Serre and Aspromonte Massifs) only pre-Triassic continental crustal units are present.

The Peloritani Mountains consist of a set of south-verging nappes of Variscan basement rocks with metamorphic grade increasing towards the top with interposed fragments of Mesozoic–Cenozoic sedimentary covers (Atzori and Vezzani 1974; Lentini and Vezzani 1975). They belong to two complexes (Fig. 1): (1) the Lower Domain, exposed in the southern part of the Peloritani Belt, consisting of very low-grade metamorphic volcano-sedimentary Cambrian–Carboniferous sequences, covered by Mesozoic–Cenozoic sediments, (2) the Upper Domain, in the north–east part of the belt, consisting of greenschist to amphibolite facies metamorphic rocks in which Aspromonte–Peloritani unit represents the highest tectonic unit. In Fig. 1 the distribution of the continental crust domains under study in this paper is mapped together with the sample location. The garnet-biotite gneiss sample derives from the Alpine tectonic mélange at the Calabria–Lucania boundary (Pollino Massif insert Fig. 1).

We describe the geological and petrological features of Mandatoriccio complex (Langone 2008), Castagna unit (Micheletti et al. 2007), high-grade metamorphic complex in Serre massif (Fornelli et al. 2011a) and Aspromonte–Peloritani unit in Calabria and Sicily (Appel et al. 2011). All these domains represent portions of middle and lower continental Variscan crust. They show Variscan evolution under decreasing P and more or less intense overprint of Alpine tectonism. Garnet–biotite gneiss sample represents a sliver of Variscan continental crust rocks included in an Alpine mélange (Laurita et al. 2015). The samples were evaluated with the aim to reconstruct the geological events recorded by the older age spectra of their zircon grains. The mineralogical composition of the samples and the respective spectra of U–Pb zircon ages are reported in Table 1.

**Table 1:** U–Pb concordant and subconcordant data on zircon in the studied rocks from CPT continental crust and Pollino Massif

	Mineralogical compositions	Older ages	Neoproterozoic-cambrian magmatism	ordovician-silurian ages	Devonian-lower permian ages	Post lower permian ages
High grade metamorphic complex						
Calabria						
GO 100 augen gneiss (Micheletti et al. 2007)	Qtz+Kfs+Pl+Bt+/-Ms+/-Grt+/-Sil	2502 ± 19, 2404 ± 92, 1760 ± 46, 752 ± 6, 617 ± 23, 575 ± 4, 572 ± 6, 571 ± 4	552 ± 9, 545 ± 4, 539 ± 7, 537 ± 4	494 ± 14, 462 ± 7		
Tur 3 restitic metagreywacke (Micheletti et al. 2008)	Grt+Pl+Opx+Amph+Bt	595 ± 12		483 ± 9	325 ± 9, 316 ± 9,308 ± 9, 297 ± 4 (n = 4), 275 ± 8	257 ± 7
GO 182 migmatitic metapelite (Micheletti et al. 2008)	Qtz+Pl+Kfs+Sil+Bt+Grt+/-Crd	*654* ± *15* ***1113*** ± ***10***		*496* ± *11*	395 ± 9 280 ± 2 (n = 18)	
Tur 17 felsic granulite (Micheletti et al. 2008)	Qtz+Pl+Kfs+Grt+/-Bt	*1688* ± *36*	*585* ± *9*		329 ± 14 286 ± 4 (n = 8)	249 ± 4
Tur 76A mafic granulite (Muschitiello 2013)	Pl+Opx+Grt+Amph+Bt		513 ± 9	466 ± 15, 436 ± 15, 434 ± 6, 413 ± 6	345 ± 4, 298 ± 10, 295 ± 9, 291 ± 6, 285 ± 17, 278 ± 6	
Grt3 mafic granulite (Fornelli et al. 2014)	Pl+Grt+Bt+Opx+Qtz+Kfs				357 ± 11, 334 ± 12-300 ± 9 (n = 8)	
MFS 3 metagabbro (Micheletti et al. 2008)	Pl+Amph+Opx+Cpx		584 ± 24, 506 ± 21	453 ± 19	377 ± 5 282 ± 5 (n = 4)	263 ± 8, 231 ± 5
Tur 49 meta-quartz-diorite (Fornelli et al. 2011b)	Pl+Opx+Cpx+Amph	744 ± 20	574 ± 18	457 ± 13, 438 ± 13	380 ± 11 347 ± 3 (n = 10) 319 ± 3 (n = 7) 296 ± 4 (n = 5)	
Tur 32 metabasite interleaved with felsic granulites (Fornelli et al. 2011b)	Opx+Pl+Bt		593 ± 14, 564 ± 17	483 ± 12, 464 ± 12, 451 ± 11, 418 ± 14	370 ± 6 (n = 3) 340 ± 7 (n = 2) 321 ± 3 (n = 9) 300 ± 3 (n = 6) 279 ± 8, 277 ± 7	260 ± 6, 252 ± 8
Tur 46 metabasite interleaved with migmatitic metapelites (Fornelli et al. 2011b)	Opx+Pl+Bt+Amph	609 ± 29	537 ± 15, 505 ± 11		382 ± 9 318 ± 5 (n = 2) 303 ± 4 (n = 4) 294 ± 4 (n = 3) 279 ± 10	
Pollino Massif						
Garnet–biotite gneiss (Laurita et al. 2015)	Pl+Qtz+Grt+Bt	1789 ± 31, 1779 ± 31, 1111 ± 44–836 ± 19 (n = 12), 701 ± 24, 696 ± 17, 610 ± 16	586 ± 17-513 ± 17 (n = 6)	475 ± 16, 457 ± 12	303 ± 8-280 ± 11 (n = 5)	255 ± 11
Castagna Unit						
GO 6 augen gneiss (Micheletti et al. 2007)	Qtz+Kfs+Pl+Bt+/-Ms	2216 ± 56, 748 ± 6, 621 ± 5, 585 ± 5	562 ± 5, 556 ± 5, 548 ± 5, 547 ± 4,543 ± 4, 542 ± 5, 541 ± 7, 515 ± 10	464 ± 4		
GO 35 augen gneiss (Micheletti et al. 2007)	Qtz+Kfs+Pl+Bt+/-Ms	2069 ± 52, 588 ± 17, 566 ± 16	556 ± 16, 552 ± 16, 544 ± 16			
GO 39 fine grained leucocratic gneiss (Micheletti et al. 2011)	Qtz+Kfs+Pl+Ms+/-Bt	858 ± 17, 632 ± 15, 631 ± 16	533 ± 11 (n = 4)	473 ± 14, 459 ± 10, 413 ± 9	302 ± 12, 302 ± 8, 296 ± 9, 294 ± 8, 287 ± 9, 286 ± 7, 285 ± 7, 282 ± 7, 281 ± 7, 275 ± 8, 275 ± 7 (n = 2), 274 ± 8	265 ± 6, 261 ± 6, 259 ± 11
GO 95 fine grained leucocratic gneiss (Micheletti et al. 2011)	Qtz+Pl+/-Ms+/-Kfs+/-Bt	801 ± 19, 633 ± 14	547 ± 3 (n = 4), 521 ± 12, 509 ± 14, 504 ± 12, 494 ± 14	452 ± 13, 437 ± 10, 425 ± 11	345 ± 9	259 ± 4 (n = 6)
Mandatoriccio Complex						
LL61b2 micaschist (Langone 2008)	Bt+Grt+And+St+Ms/-Crd+/-Sil	2506 ± 43–604 ± 24 (n = 30)	587 ± 14–511 ± 13 (n = 8)	485 ± 13–428 ± 10 (n = 8)		
*VSerre Massifariscan granitoids*						
Tur 37b Quartz-monzodiorite dike (Fornelli et al. 2011b)	Kfs+Bt+Opx+Cpx+Qtz				368 ± 11, 367 ± 9 323 ± 5 (n = 3)	
Upper domain Aspromonte–Peloritani unit						
Calabria						
ADR 5 augen gneiss (Micheletti et al. 2007)	Qtz+Kfs+Pl+Bt+/-Ms	917 ± 26, 614 ± 10, 611 ± 11, 597 ± 10, 586 ± 10	577 ± 10, 568 ± 10, 566 ± 13, 550 ± 16, 527 ± 12			
ADR 18 augen gneiss (Micheletti et al. 2007)	Qtz+Kfs+Pl+Bt+/-Ms	623 ± 18, 617 ± 17, 565 ± 16	548 ± 16, 531 ± 15, 526 ± 15, 522 ± 15	446 ± 13		
Peloritani						
FIU-7 paragneiss (Williams et al. 2012)	Qtz+Bt+Pl+/-Grt+/-Sil	2672 ± 9–611 ± 6 (n = 46)	566 ± 15–535 ± 4 (n = 14)			
FIU-11 augen gneiss (Williams et al. 2012; Fiannacca et al. 2013)	Qtz+Kfs+Pl+Bt+/-Ms+/-Sil	2627 ± 25–607 ± 5 (n = 12)	578 ± 10–516 ± 4 (n = 23)			
MV-15 augen gneiss (Williams et al. 2012; Fiannacca et al. 2013)	Kfs+Qtz+Pl+Bt+/-Ms	2455 ± 9–634 ± 14 (n = 12)	557 ± 6–528 ± 7 (n = 25)			
TC-9 augen gneiss (Fiannacca et al. 2013)	Kfs+Qtz+Pl+Bt		581 ± 3–528 ± 4 (n = 35)			
Lower domain						
Peloritani						
Ta-Pu1-2-3, Ta-Cs, Tao Felsic porphyroids and andesites (Trombetta et al. 2004) *ID* - *TIMS data*	Qtz+Kfs+/-Bt+/-Ms+/-Chl	2013 ± 1, 1140 ± 10		461 ± 10–432 ± 15 (n = 16)	401 ± 20, 367 ± 13	

Mean concordia ages are indicated in underline

Discordant data (in this case ^206^Pb/^238^U data have been considered) are indicated in italic

An upper intercept in GO182 sample is indicated in italic bold

*Qtz* quartz, *Pl* plagioclase, *Kfs* K-feldspar, *Grt* garnet, *Bt* biotite, *Ms* muscovite, *Amph* amphibole, *Crd* cordierite, *Opx* orthopyroxene, *Cpx* clinopyroxene, *Sil* sillimanite, *And* andalusite, *St* staurolite

### Castagna unit

The Castagna Unit underlies the high-grade metamorphic complex; it consists of paragneisses, micaschists, augen gneisses, Variscan granitoids and minor amphibolites, quartzites, Ca-silicate rocks and marbles (Colonna and Piccarreta 1976; Paglionico and Piccarreta 1976). It is exposed in the Sila and Serre Massifs (Fig. 1) and includes rocks equilibrated under greenschist to amphibolite facies conditions in Variscan times and reworked by Alpine tectonics (Colonna and Piccarreta 1976; Langone 2008; Micheletti et al. 2007, 2011). The magmatic protoliths of augen gneisses were intruded in the metasediments of Castagna Unit.

### Mandatoriccio complex

The Mandatoriccio Complex is exposed in the Sila Massif (Fig. 1). It is tectonically overimposed on high-grade deep crustal rocks and consists of medium-grade metapelites, meta-arenites, meta-volcanites and metabasites with rare marbles and orthogneisses (Acquafredda et al. 1988, 1991; Langone 2008). Micaschists show a static porphyroblastic growth of biotite, garnet, andalusite, staurolite, muscovite and minor cordierite and fibrolite (Lorenzoni and Zanettin-Lorenzoni 1979; Borghi et al. 1992; Langone 2008). A clockwise P–T–t path with a metamorphic peak at about 590 °C and 0.35 GPa during Variscan post-orogenic extension dated 299 Ma has been defined (U–Th–Pb monazite ages; Langone et al. 2010).

### High-grade metamorphic complex in Serre

**Fig. 2:**
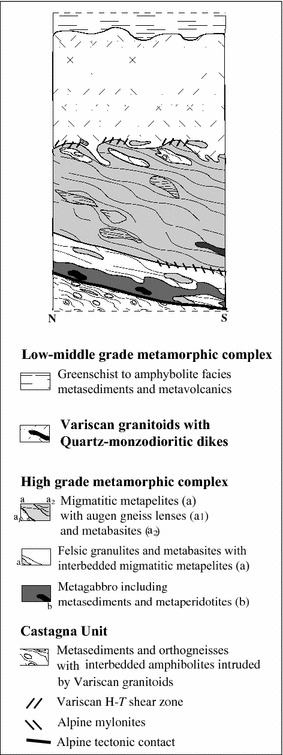
Schematic section (not in scale) of the continental Variscan crust in the Serre massif (modified from Fornelli et al. 2011a)

This complex occupies wide areas in the Sila and Serre Massifs (Fig. 1). In the Serre massif, an about 20 km thick section is exposed (Schenk 1989; Acquafredda et al. 2003). It is formed by 7–8 km thick lower crustal rocks equilibrated under granulite–amphibolite facies conditions underlying low-middle grade metamorphic complex of the upper crust (Fig. 1). Both upper and lower crustal domains were intruded by 10 km-thick “layer” of Variscan granitoids (Fig. 2) emplaced about 300 Ma ago (Schenk 1980; Caggianelli et al. 2000).

The Variscan lower crust of the Serre includes, from the bottom (Fig. 2): (a) felsic granulites, metagabbros, metabasites, rare meta-peridotites, metagreywackes and metapelites, (b) migmatitic metapelites representing the wide portion of the Serre Massif (Schenk 1984; Fornelli et al. 2002), with interleaved metagreywackes, metabasites, rare marbles and augen gneisses. The augen gneisses preserve original intrusive features of their protoliths in the metasediments. The lower crustal rocks have been affected by pervasive partial melting (Maccarrone et al. 1983; Schenk 1984; Caggianelli et al. 1991; Fornelli et al. 2002) mostly during the Late Carboniferous–Permian exhumation (Fornelli et al. 2002). Thermobarometric calculations related to the Serre rock types (Acquafredda et al. 2006, 2008; Fornelli et al. 2011b) give: (1) T-peak of ~700 °C and of ~900 °C and P-peak of 0.9 and ~1.0–1.1 Gpa at the top and bottom of the section respectively, and (2) T-peak followed by quasi-isothermal decompression of about 0.5–0.6 Gpa at the top and by total decompression of about 0.3 Gpa at the bottom during Late Carboniferous—Permian times (Fornelli et al. 2011a). During the crustal thinning, at about 323 ± 5 Ma (Fornelli et al. 2011a) some quartz-monzodioritic dikes were emplaced in the middle part of the metapelites (Schenk 1984), subsequently, at about 300 Ma ago (Schenk 1980; Caggianelli et al. 2000), huge volumes of calc-alkaline granitoids were emplaced between the low-middle and high-grade metamorphic complexes (Fig. 2).

### Aspromonte–Peloritani unit

In the Aspromonte Massif (Southern Calabria), this Unit is sandwiched between the lower “Madonna di Polsi” Unit (Pezzino et al. 2008) not mapped in Fig. 1 and the super-imposed low-grade metamorphic complex, whereas, in the Peloritani massif, it is the highest tectonic Unit. The prevalent rock types are middle-grade biotite paragneisses and augen gneisses (derived from intruded granitoids) with minor amphibolites, micaschists and marbles (Fig. 1). The metamorphic rocks are extensively intruded by late-Variscan peraluminous granitoids (D’Amico et al. 1982; Rottura et al. 1990, 1993; Fiannacca et al. 2005, 2008). The Aspromonte–Peloritani Unit appears as the product of processes of crustal thickening during early- and middle-Variscan collisional stages, followed by crustal thinning, granitoid intrusion and unroofing during late-Variscan extensional stages (Festa et al. 2004; Caggianelli et al. 2007).

U–Pb monazite ages from paragneisses of the Aspromonte Massif dated ~300 Ma the metamorphic peak under T max of 620 °C and P max of ca. 0.25 Gpa (Graeßner et al. 2000; Appel et al. 2011). This metamorphic peak was nearly synchronous with the granitoid intrusions at 303–290 Ma (Appel et al. 2011 and references therein).

## Analytical procedures

Zircon age determinations in samples Tur 3, Go 182, Tur17, Tur 76A, Grt3, MFS 3, Tur 49, Tur 32, Tur 46, garnet–biotite gneisses, GO 39, Go 95, LL61b2 and Tur 37b (Table 1) were performed using a 193 nm ArF excimer laser-ablation (LA) microprobe (GeoLas200QMicrolas) coupled to a magnetic sector ICP-MS (inductively coupled plasma-mass spectrometer; Element 1 from Thermo Finnigan) at IGG-CNR (Pavia, Italy). The analytical procedures to acquire, collect and process data are reported in Fornelli et al. (2011b).

Zircon ages in samples GO 100, Tur 17, GO 6, GO 35 ADR 5 and ADR 18 (Table 1) were carried out using a Cameca SIMS-1270 ion microprobe (CRPG-CNRS of Nancy, France). Details on data acquisitions are reported in Micheletti et al. (2007).

Zircon ages in samples FIU-7, FIU-11, MV-15 and TC-9 (Table 1) were performed on the ANU SHRIMP II ion microprobe using procedures based on those described by Williams and Claesson (1987). The operative procedures are described in Williams et al. (2012) and Fiannacca et al. (2013).

As regards the samples Ta–Pu 1, Ta–Pu 2, Ta–Pu 3, Ta–Cs and Tao, the zircon ages were acquired using a Finnigan-MAT 262 multicollector thermal ionization mass spectrometer calibrated against NBS 982+U500 at Geological and Mineralogical Museum of Oslo. Details are reported in Trombetta et al. (2004).

The analytical data of U–Pb zircon ages of considered samples are reported in Additional file 1 except for age data derived from Langone (2008), Laurita et al. (2015) and Trombetta et al. (2004) which should be referred.

The concordia test was performed for each analytical spot from 206Pb/238U and 207Pb/235U ratios using the function in the software package Isoplot/Ex3.00 (Ludwig 2003). The same software was used to calculate the Mean Concordia Age, the Mean Square of Weighted Deviates (MSWD) and the probability of concordance.

Trace element compositions on zircons were collected by LA-ICP-MS (CNR—Istituto di Geoscienze e Georisorse Unità di Pavia, Italy). Details of procedures are in Fornelli et al. (2011b).

## Chronology of zircon

The zircon domains from twenty-four samples here considered, show various spectra of ages having different geological significance (Table 1). The majority considered ages have a probability of concordance >75 %.

Variscan zircon domains occur and are decidedly abundant only in the granulite facies metasediments and metabasites of the lower crust of the Serre and in garnet–biotite gneiss from Pollino massif, whereas they are absent in the augen gneisses and metasediments of the Aspromonte–Peloritani Unit and Mandatoriccio Complex (Table 1).

Ordovician–Silurian domains characterize the zircons from Calabria (Aspromonte and Castagna) augen gneisses, deep crustal rocks of the Serre, garnet–biotite gneiss of Pollino and metasediments of Mandatoriccio complex. However, in the Mandatoriccio complex and in garnet–biotite gneiss from Pollino, the Ordovician–Silurian ages are related to detritic grains (Langone et al. 2010; Laurita et al. 2015), whereas in the Calabria augen gneisses and deep crustal rocks they have been interpreted as resetted or recrystallized domains (Fornelli et al. 2011a).

Lower Cambrian–pre-Cambrian zircon ages are present, in different proportions, in all considered rocks. It has to be noticed that these ages are abundant in the garnet–biotite gneiss from Pollino massif while have been mostly erased in the higher-grade deep crust rocks of the Serre massif. In the following sections the significance of the age clusters in the examined rocks is discussed.

### Augen gneisses

**Fig. 3:**
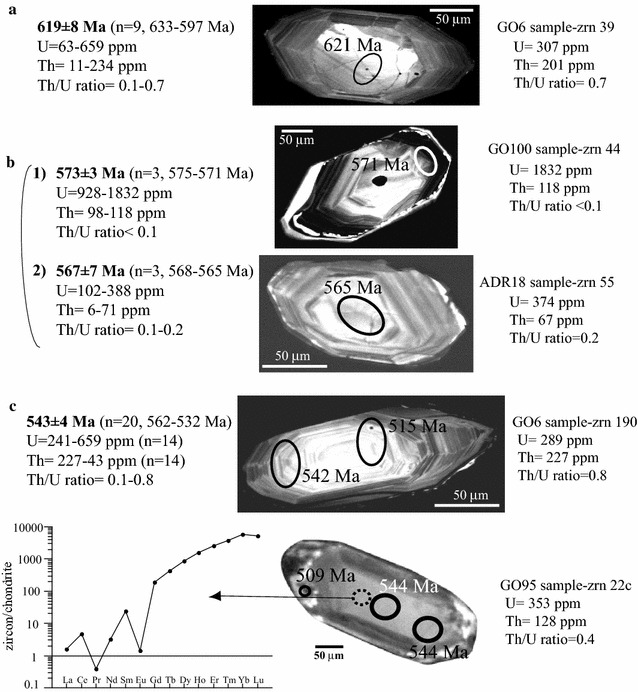
Selected zircon crystals of augen gneisses showing internal structures and spot ages. Statistically significant cluster U–Pb ages, Th–U contents and Th/U ratios are also indicated in **a**, **b** and **c**. In **c** REE pattern of zircon dated 544 Ma is reported (*dotted circle* indicates the analysed site). SEM images (cathodoluminescence detector) and data ages of zircons derive from Micheletti et al. (2007)

The augen gneisses from Calabria contain zircon inherited grains giving ages ranging from 2502 to 1760 Ma as clusters or single spots; six ages in the range 748–917 Ma (Table 1) and a lot of the concordant ages ranging from 633 to 532 Ma (Micheletti et al. 2007, 2011). The last age group defines statistically significant clusters (Table 1; Fig. 3): (a) at 619 ± 8 Ma (mean concordia age of nine ages ranging from 633 to 597 Ma) relative to cores having variable U (63–659 ppm) and Th (11–234 ppm) contents and Th/U ratios significantly higher than 0.1 (0.1–0.7), (b) from 575 to 565 Ma including three rims (1 in Fig. 3b) with high U contents (928–1832 ppm) and two core domains and one rim (2 in Fig. 3b) with low U (102–388 ppm), Th ranging from 6 to 71 ppm and Th/U in the range 0.1–0.2 and (c) at 543 ± 4 Ma (20 ages 562-532 Ma, Fig. 3c) mainly related to rims with high Th/U ratio (0.1 to 0.8).

The first age cluster (619 ± 8 Ma) includes rounded or fractured cores appearing as detrital domains (Fig. 3a). Accordingly, the zircon domains averaging 619 ± 8 Ma and the older ones are to be considered as inherited from the source material (Fig. 3a). The three rims dated from 575 to 571 Ma (mean concordia age 573 Ma in Fig. 3b) with high U contents and low and quite similar Th/U ratios (≤0.1) seem to imply that Th and U contents at the time of the zircon growth were probably controlled by the same reactions and suggest compatibility with a metamorphic origin (Rubatto and Hermann 2007; Xia et al. 2009), as well as the cluster at 567 Ma with lower Th/U ratio (Fig. 3b). The cluster peaking at 543 Ma (n = 20) includes many euhedral crystals showing continuity between core and rim having high U contents (14 spot ranging from 659 to 241 ppm) and Th/U ratios mostly between 0.2 and 0.5 (Fig. 3c); one domain analysed for REEs produces a highly fractionated pattern and a distinct negative Eu anomaly (Fig. 3c). The characteristics of this population are common to magmatic zircons (Rubatto and Hermann 2007) or to recrystallised domains preserving memory of parental magmatic zircons (Xia et al. 2009). The moderate variability and the high values of Th/U seem to be consistent with precipitation from a hybrid magma precursor of the augen gneisses (Fornelli et al. 2007).

The augen gneisses from Peloritani contain zircon grains giving ages ranging from 3200 to 520 Ma as clusters or single spots (Williams et al. 2012; Fiannacca et al. 2013).

**Fig. 4:**
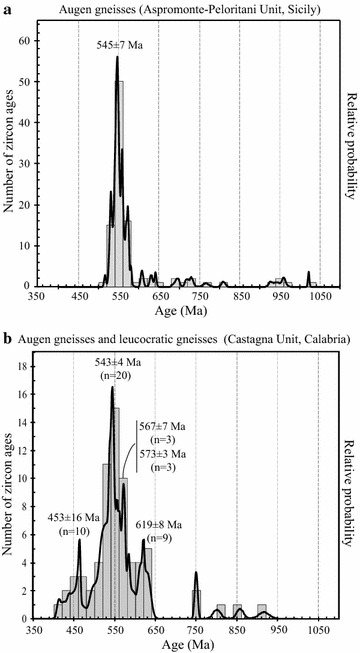
Histograms and probability density curves of concordant zircon ages from augen gneisses of Aspromonte–Peloritani (**a**) and Castagna Units (**b**) are reported (data from Fiannacca et al. 2013; Micheletti et al. 2007)

Many zircon domains are younger than 630 Ma and form a significant cluster at ≈545 Ma (Fig. 4a) including two kinds of zircon domains having U contents of 320–940 and 40–470 ppm interpreted as suggestive of magmatic and detrital origin, respectively (Williams et al. 2012). The age spectrum, on the whole, overlaps that related to Calabria augen gneisses (Fig. 4b) apart from (1) the absence, except for one domain (917 Ma), of ages 900–1000 Ma in augen gneisses from Calabria (Fig. 4b) and (2) the lack of Ordovician–Silurian ages in augen gneisses from Peloritani (Fig. 4a) and (3) the presence of a relevant peak at 453 Ma in augen gneisses of Calabria (Fig. 4b).

### Metabasic rocks from the lower crust

**Fig. 5:**
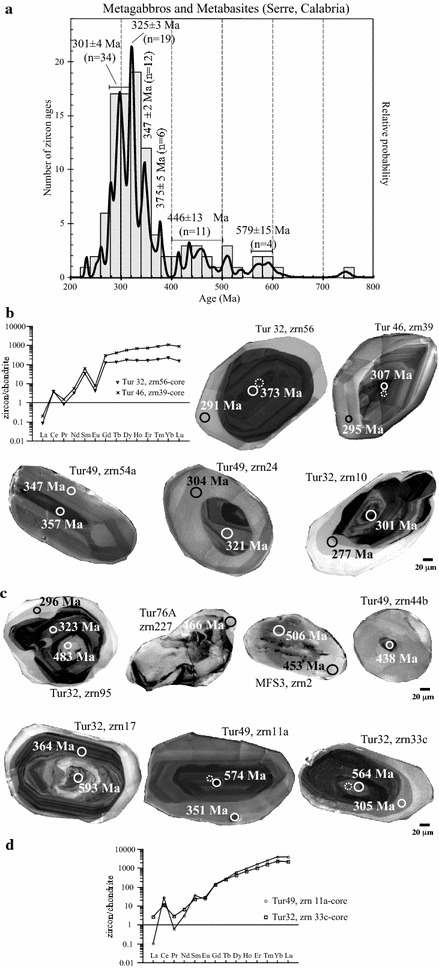
Histograms and probability density curves of concordant zircon ages from metagabbros and metabasites of Serre are shown (**a**). SEM images (VPSED, Variable Pressure Secondary Electron Detector) of dated zircons from metagabbros and metabasites of Serre are reported in **b**, **c** and **d**. REE patterns of metamorphic (**b**) and magmatic (**d**) zircons were also shown. *Dotted circles* indicate the sites of REE analyses. (data from Micheletti et al. 2008; Fornelli et al. 2011a; Muschitiello 2012)

Zircons from metagabbros and metabasites interbedded with felsic granulites and migmatitic metapelites in the Serre have been considered (Micheletti et al. 2008; Fornelli et al. 2011a). The most of analyses on separates and on thin section produce ages (Table 1; Fig. 5a) mostly between 373 and 277 Ma (Fig. 5a, b), single Ordovician–Silurian spots (446 ± 12 Ma; Fig. 5a–c;) and Neoproterozoic ages (in average 579 Ma, Fig. 5a and from 564 to 593 Ma Fig. 5d). A first hand interpretation of this data would lead to propose that this basic magmatism occurred in the Variscan times. However:

1. many Variscan domains of zircon (Fig. 5b) from garnet-bearing rock types show evidence of the “garnet effect” in a closed system, such as flat HREE patterns (Fornelli et al. 2011a), in contrast with domains precipitated from a melt (Rubatto 2002). Thus the above domains formed or recrystallised in presence of garnet, which is metamorphic in origin (Fornelli et al. 2011a);
2. many Ordovician–Silurian domains (Fig. 5c) form rims around older cores (Micheletti et al. 2007). Eleven ages form a cluster at 446 Ma in the range 483–413 Ma (Fig. 5a);
3. Eight ages in the range 505–593 Ma include four domains dated 593–564 Ma (in average 579 ± 15 Ma Fig. 5a–d) showing oscillatory zoning, high Th/U ratios (0.16–0.19) and fractionated REE patterns (Fig. 5d; Fornelli et al. 2011a), these features are compatible with a magmatic origin (Rubatto 2002).

On this basis, it seems that the mafic magmatism occurred in Neoproterozoic time (579 Ma), some tens of million years earlier than the felsic magmatic precursor of the augen gneisses at 543–545 Ma (Micheletti et al. 2008; Fornelli et al. 2011a, Fornelli et al. 2012; Williams et al. 2012). It is noteworthy that Neoproterozoic-Lower Cambrian felsic and mafic magmatism is recorded in many of the so-called “Cadomian blocks” present from the Iberia, Pyrenees, Western Alps to Turkey (e.g. Neubauer 2002; Stedra et al. 2002; Castiñeiras et al. 2008; Fernàndez-Suarez et al. 2013).

### Metasedimentary rocks

**Fig. 6:**
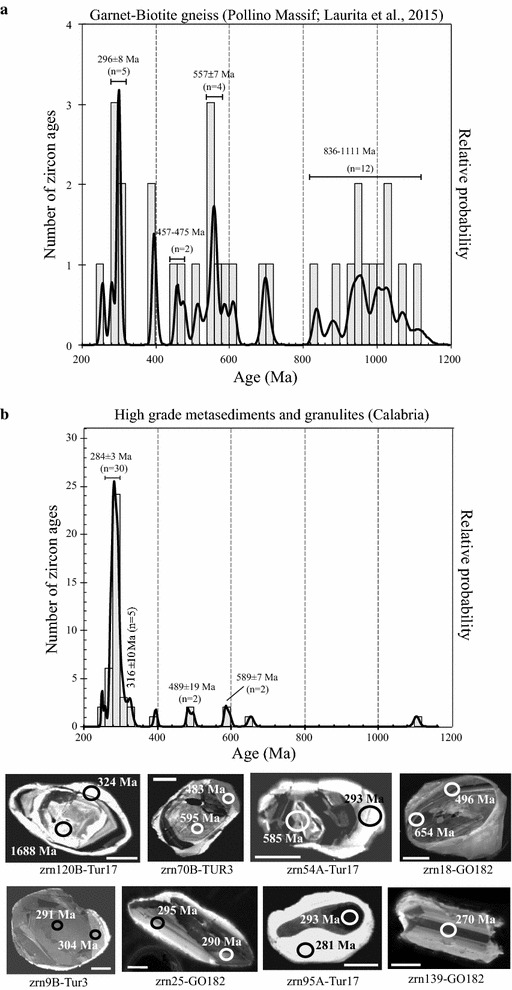
Histograms and probability density curves of concordant zircon ages from garnet–biotite gneisses (**a**) from continental crust sliver of Pollino massif (data from Laurita et al. 2014) and high-grade metasediments of Serre (**b**; data from Fornelli et al. 2011a). In (**b**) SEM images of dated zircons from high grade metasediments (from Micheletti et al. 2008) are shown (*scale bar* 50 μm)

U–Pb zircon ages of garnet–biotite gneiss from Pollino massif were considered. This gneiss shows features of high-grade metamorphism and represents a sliver of Calabria lower continental crust in the Alpine mélange (Laurita et al. 2015). The zircon age data span from 1789 to 255 Ma (Table 1) with age cluster (Fig. 6a) in the range 836–1111 Ma (n = 12) and some data in each cluster at 610–696, 513–586 Ma (in average 557 ± 7 Ma), 457–475 and 296 ± 8 Ma, (Laurita et al. 2015). The Ordovician to pre-Cambrian ages were related to heritages whereas Carboniferous–Permian ages were related to the Variscan metamorphism.

U–Pb data on zircon grains are available (Table 1) for migmatitic metapelites (sample GO 182), restitic metagreywacke (Tur 3) and felsic granulites (sample Tur 17) from the Serre, paragneisses from the Peloritani (sample FIU-7) and micaschists from the Mandatoriccio Complex in Sila (sample LL61b2) (Micheletti et al. 2008; Langone 2008; Fornelli et al. 2011a; Williams et al. 2012). Micheletti et al. (2008) report a few Neoproterozoic inherited zircon ages (^206^Pb/^238^U ages 585, 595, 654 and 1688 Ma) from the granulite facies metasediments of the Serre, and many ages in the range 325–270 Ma (Table 1; Fig. 6b). Three of the inherited ages are discordant evidencing Pb loss during the long geological history. In addition, an upper intercept at 1113 ± 100 Ma (Table 1) from discordant data was calculated for the migmatitic metapelite (Micheletti et al. 2008). The age data distribution in the high-grade metasediments from the Serre shows significant age peaks (Fig. 6b) interpreted as: (1) Variscan metamorphism (284 Ma), (2) memory of Ordovician–Silurian activity (489 Ma) and signatures of mafic magmatism (589 Ma) as discussed before (Figs. 5a, 6b). In garnet–biotite gneiss from Pollino Massif similar age peak distribution can be observed apart from the significant age cluster at 1111–836 Ma (Fig. 6a).

**Fig. 7:**
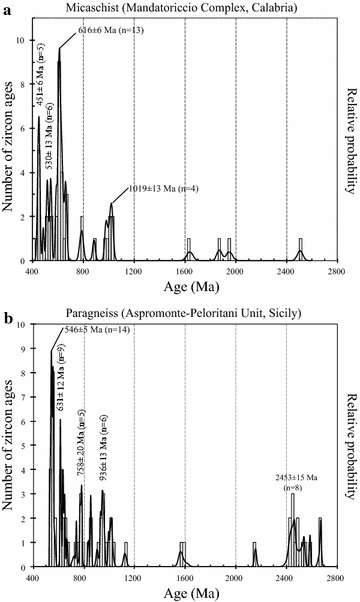
Histograms and probability density curves of concordant zircon ages from micaschists and paragneisses of Mandatoriccio complex (**a**) and Aspromonte–Peloritani Unit (**b**) (data from Langone 2008, Williams et al. 2012)

Zircons from the amphibolite facies metasediments of Aspromonte–Peloritani Unit in Sicily (Williams et al. 2012) and Mandatoriccio complex in Calabria (Langone 2008) preserve significant pre-Variscan (>400 Ma) memory (Fig. 7; Table 1). The Neoproterozoic ages of the Mandatoriccio Complex (Fig. 7a) show strong similarities with the ages from the paragneisses of Aspromonte–Peloritani Unit (Langone 2008; Williams et al. 2012): significant age peaks between 700 and 500 Ma, some ages comprised between 1000 and 750 Ma (Fig. 7) and minor older age data (>1600 Ma). The Mandatoriccio micaschists, however, preserve also a lot of detrital zircon grains (Langone 2008) having Ordovician–Silurian ages (451 ± 6 Ma; Fig. 7a) absent in paragneisses of Aspromonte–Peloritani Unit (Fig. 7b). In the low-grade metasediments from both Calabria and Sicily, Ordovician–Silurian ages for sedimentation and magmatism have been indicated (Trombetta et al. 2004; Martín-Algarra et al. 2014) and zircon separates dated around 500 and 2000 Ma were recorded in Serre (Schenk and Todt 1989).

## Sm-Nd isotopic features

Augen gneisses, metabasites and high-grade metasediments have been chemically analysed (Moresi et al. 1979; Fornelli et al. 2002, 2007; Muschitiello 2013; Fiannacca et al. 2013). The felsic protoliths of the augen gneisses were alkali-calcic hybrid magmas dominated by crustal components (Fornelli et al. 2007). The mafic-intermediate protoliths of metabasites were mantle-derived calc-alkaline magmas more or less crustal contaminated (Micheletti et al. 2007; Fornelli et al. 2007). The high-grade metapelites are deeply restitic after the extraction of about 40 % of “granitic melts” during the Variscan orogenesis (Fornelli et al. 2002).

From a geodynamic point of view the bimodal Neoproterozoic–Cambrian magmatism seems to be related to an active continental margin (Fornelli et al. 2007).

The Nd model ages (at 540 Ma) of the Calabria augen gneisses range from 1700 to 1530 Ma (Micheletti et al. 2007) and are decidedly older than the U–Pb zircon ages (Table 2). This provides evidence for an origin by melting or re-melting of an older crust (Fornelli et al. 2007). The ε_Nd (562–526 Ma)_ values (Table 2) in augen gneisses range from −3.19 to −5.35 (Micheletti et al. 2007).

**Table 2:** ε_Nd_ and Nd t_DM_ (Ma) values in different rock types of Castagna (*CU*), Sila (*SU*) and Aspromonte–Peloritani Units (*APU*)

Rock tipology	Source	ε_Nd_	Nd t_DM_ (Ma)	Reference age (Ma)
Augen gneisses (*CU*, n = 5)	Micheletti et al. (2007)	−3.19/−5.35	1530–1700	526–562
High grade metasediments and granulites (*SU*)	Caggianelli et al. (1991)	−7.5/−14.7	1800–2400	550
High grade metasediments and granulites (*SU*)	Schenk (1990)		1350	
Medium-high grade paragneiss (*APU*, n = 1)	Williams et al. (2012)	−6.6	1750	540
Augen gneisses (*APU*, n = 3)	Fiannacca et al. (2013)	−3.21/−4.45	1520–1600	545–565

The augen gneisses from Peloritani have Nd model ages (1600–1520 Ma) and ε_Nd_ values (from −3.21 to −4.45) both calculated at 565 and 545 Ma (Fiannacca et al. 2013) very similar to those calculated for the Calabria augen gneisses (Table 2).

Nd model ages in the granulite facies metasediments of the lower crust of the Serre Massif show a wider data range from 1350 to 2400 Ma (Schenk 1990; Caggianelli et al. 1991) and the ε_Nd (550 Ma)_ values (−7.5 and −14.7) are lower than in the augen gneisses (Table 2). The paragneiss from Peloritani gives Nd model age of 1750 Ma and ε_Nd (540 Ma)_ value of −6.6 (Williams et al. 2012).

## Geological inferences

### Deposition age of crustal source of the augen gneisses

The Calabria augen gneisses relate to hybrid magmas (Fornelli et al. 2007) emplaced into metasediments around 543 ± 4 Ma (Fig. 4b; Micheletti et al. 2007, 2011). They contain Neoproterozoic to Archean inheritance represented by rounded and fractured zircon cores interpreted as detritic (Micheletti et al. 2007). A representative population (13 %) forms a statistically significant cluster peaking at 619 ± 8 Ma (Figs. 3a, 4b) of domains having variable Th/U ratios (0.1–0.7), which is followed by another significant cluster at 573 ± 3 Ma (Figs. 3b, 4b) calculated on rims having low and homogeneous Th/U ratios (≤0.1, Fig. 3b). The latter cluster is interpreted as indicative of the time of metamorphic zircon growth and imposes an absolute limit to the sedimentation that should be older than 573 ± 3 Ma. Considering that the older mean concordia age is 619 ± 8 Ma (Fig. 4b) than this age could approximate the sedimentation age of the protoliths.

The span of time between the presumed age of the sedimentation (619 ± 8 Ma) and the magmatic crystallization ages (in average 543 ± 4 Ma in Fig. 4b) of protoliths of augen gneisses might account for the evolution from sedimentation, metamorphism to partial melting stages during the Cadomian orogenesis.

This reconstruction does not agree with that hypothesized for the equivalent augen gneisses from Peloritani massif. Williams et al. (2012) and Fiannacca et al. (2013) envisage an almost synchronous process from sedimentation to partial melting (at 545 ± 7 Ma Fig. 4a and 546 ± 5 Ma in Fig. 7b) of the paragneisses hosting the augen gneisses because they measured similar zircon ages both in paragneisses and augen gneisses (Figs. 4a, 5, 6, 7b). In the proposed geological model for the Peloritani area, however, the evidences of restitic features of paragneisses compatible with extraction of abundant melt in Neoproterozoic–Cambrian times have not yet been documented. In addition, the ε_Nd_ values calculated for Calabria augen gneisses (from −3.19 to −5.35 in Micheletti et al. 2007) are higher than ε_Nd_ of the Peloritani paragneiss (−6.6 in Williams et al. 2012) precluding a direct link. We think that the similar age distribution in augen gneisses and paragneiss of Peloritani (Figs. 4b, 5, 6, 7b; Table 1) could be due to rejuvenation of zircon from paragneisses caused by intruding magmas (protoliths of augen gneisses), or by younger tectono-thermal events (Ordovician and Variscan) well documented in the felsic porphyroids and andesites from Peloritani (Table 1; e.g. Trombetta et al. 2004; Appel et al. 2011). The hypothesized metamorphism in Neoproterozoic–Cambrian times in Calabria at about 573 Ma (this paper) and in Peloritani paragneiss around 535 Ma (Williams et al. 2012; Fiannacca et al. 2013) give information about the evolution of Panafrican/Cadomian orogenesis.

### Depositional ages of protoliths of the metasediments

The metasediments of the Serre, Castagna and Aspromonte–Peloritani terrains were intruded in Neoproterozoic times by acidic (543–545 Ma) and basic (579 ± 15 Ma) magmas (Micheletti et al. 2007, 2008). On this basis, the deposition of protoliths of metasediments must have been older than magma emplacements. This agrees with conclusion of Schenk (1990) indicating a sedimentation age from 1000 to 600 Ma for the high-grade metasediments of the Serre, on the basis of Sr isotopic evolution. In Variscan times these crustal domains were affected by medium- high-grade metamorphism. (Micheletti et al. 2008; Fornelli et al. 2011a). However, only the deep crustal metamorphites of the Serre and the garnet–biotite gneiss from Pollino massif evidenced zircon domains formed in Variscan times (Table 1; Fig. 6). The granulite facies conditions as well as the pervasive fluid-present dehydration melting in the Serre (Fornelli et al. 2002) seem to account for generation of new zircon and/or modification of the older ones erasing nearly completely the pre-Cambrian ages (Fornelli et al. 2011a, Fornelli et al. 2012) which, however, are present in garnet–biotite gneiss from Pollino massif (ages 1111–836 Ma Fig. 6a) where the Variscan metamorphism was not able to produce significant annealing/recrystallization processes in zircons (Laurita et al. 2015), probably as effect of lower temperatures of metamorphism. The here deduced deposition age of protoliths of metasediments (>600 Ma) is decidedly older than that suggested by Laurita et al. (2015) indicating 457 Ma as maximum depositional age for the sedimentary protoliths of garnet–biotite gneiss of the Pollino massif, which look like the high-grade metasediments of Calabria.

As concerns the Peloritani paragneisses and the Mandatoriccio micaschists representing medium grade metasediments, must be evidenced that the youngest detrital zircon age in the former was 535 ± 4 Ma (FIU-7 sample in Table 1) whereas in micaschists was in Ordovician–Silurian times 428 ± 10 Ma (LL61b2 sample in Table 1). These facts indicate the Lower Cambrian as minimum sedimentation age for Peloritani paragneisses (Williams et al. 2012) and the Ordovician–Silurian times as maximum sedimentation age for Mandatoriccio micaschists (Langone 2008).

According to our interpretation, the geological evolution of these terrains in pre-Paleozoic times was distinct: lower and intermediate Variscan crust portions (metasediments of Serre, Castagna and Aspromonte–Peloritani) record an older history with respect to Mandatoriccio complex, sliver of garnet–biotite gneiss from Pollino and very low-grade Variscan metasediments. In fact the very low-grade metasediments in southern Calabria and Sicily contain porphyroids and meta-andesites having Ordovician ages (Acquafredda et al. 1991) and Ordovician-Silurian zircon ages were revealed in low-grade metasediments of Serre (e.g. Martìn-Algarra 2014).

## Provenance

According to Stampfli et al. (2011) the pre-Variscan basements dispersed in the Mediterranean areas were mostly derived from Gondwana supercontinent. They consist of detrital materials derived from both East and West Gondwana cratonic sources since the Neoproterozoic time (von Raumer et al. 2013).

The provenance of materials forming the pre-variscan basements might be identified on the basis of (1) age of inheritance (Mallard and Rogers 1997), (2) nature and age of the magmatism, (3) absence or presence of ages falling in specific time spans and (4) isotopic characteristics (Mallard and Rogers 1997; Linnemann et al. 2008). However, it has to be pointed out that in the case under study the source materials experienced Neoproterozoic–Cambrian to Variscan tectonothermal events (Figs. 4, 5, 6, 7). So inheritances could be affected by partial to complete resetting and the interpretation of individual ages could be misleading.

**Fig. 8:**
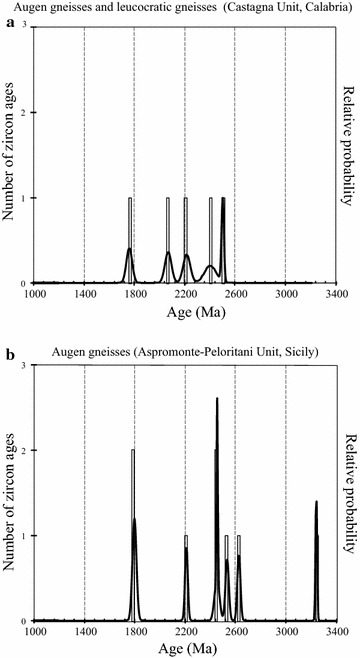
Histograms and probability density curves of inherited zircon ages in the range 1000–3400 Ma collected in the augen gneisses from Castagna (**a**) and Aspromonte–Peloritani (**b**) Units (data from Micheletti et al. 2007; Fiannacca et al. 2013)

Augen gneisses coming from Calabria and Peloritani domains are promising for the provenance analyses (Fig. 8). The relevant points that seem to reflect a West African provenance (Micheletti et al. 2007) for the metasedimentary protoliths of the granitic magmas are (Figs. 4, 5, 6, 7, 8): (1) all augen gneisses show overlapping age data and there is no evidence of direct link between the Neoproterozoic-Early Cambrian felsic magmas and the associated metamorphic rocks including the migmatites of the Serre deep crust (Figs. 4, 6b, 7b); (2) the paucity of components aging 1050–900 Ma (Fig. 4), the presence of component dated 2600–1700 Ma (Fig. 8), the gap of ages between 1700 and 1050 Ma (Fig. 8); (3) the quite homogeneous Nd model ages (1530–1700 Ma; Table 2) and the similar chemical composition of the protoliths of Calabria augen gneisses with that of granitoids from the Anti-Atlas Morocco domain (Gasquet et al. 2005; Micheletti et al. 2007). All these features lead to West African Craton provenance (Nance et al. 2008). However, according to Williams et al. (2012), the paragneisses associated with the Peloritani augen gneisses bear evidence of a East African provenance owing the similarity of the distribution ages with those of Jordan and Israel rock-types.

**Fig. 9:**
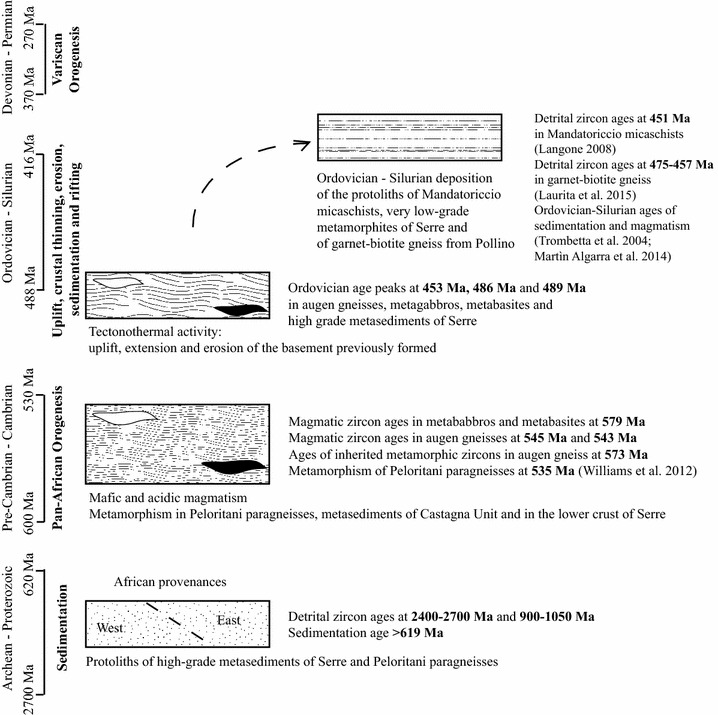
Schematic model of geological and geochronological evolution of Calabria–Peloritani Terranes from Archean to Silurian times is shown

Unfortunately: (1) Precambrian ages have been mostly erased in the granulite facies deep crustal rocks of the Serre (Fig. 6b; Micheletti et al. 2008); (2) zircon has not so far analysed (no data) in the Castagna metasediments. Speculatively, we can assume that: (a) the pre-Cambrian sediments derive from East and West African sources; (b) the Ordovician sediments reflect erosion of Pan-African orogen and local input from Grenvillian terrains as evidenced by the high concentration of zircon ages between 836 and 1111 Ma (Fig. 6a) in the sliver from Pollino massif (Laurita et al. 2015), which assumed features looking like deep crustal rocks of Calabria during the Variscan orogenesis. The provenance from peri-Gondwanan cratonic areas common to many Precambrian blocks (e.g. von Raumer et al. 2013), might be framed in the Calabria–Peloritani Terrane in the following restoration (Fig. 9): (1) blocks of West and East African provenance were amalgamated during the Pan-African orogeny, as the possible metamorphism at 573 ± 3 Ma (or 535 Ma according to Williams et al. 2012) suggests (Fig. 3b); (2) widespread Neoproterozoic-Early Cambrian bimodal magmatism with emplacement of basic and acidic protoliths of metagabbros, metabasites and augen gneisses was diffused within this amalgamated basement formed by Peloritani paragneisses and high-grade metasediments as the similarity of Precambrian records in these rocks lead to believe; (3) Ordovician tectono-thermal activity recorded in the described basement caused uplifting, rifting and erosion of these terrains as can be verified in Northern Africa paleozoic sediments (Fiannacca et al. 2008; Williams et al. 2012); (4) the derived detritus supplied the Palaeozoic basins (Langone 2008) as the pre-Silurian detrital zircons in the Mandatoriccio micaschists and in garnet–biotite gneiss of Pollino together with the Silurian ages of low-grade metasediments in Serre suggest; (5) subsequently Variscan orogenesis involved these terranes as the zircon ages comprised between ~370 and ~270 Ma in higher grade metamorphites indicated.

The lack of Ordovician–Silurian records in the Peloritani metasediments (Fig. 7b) can be due to the small number of analysed samples.

## Conclusions

From the synthesis of the available data it appears that the Peri-Gondwana Calabria–Peloritani Terrane, affected by Variscan tectono-thermal events, preserves records of previous geological processes (Fig. 9):

1. Neoproterozoic sediments derived from both West and East African cratonic sources, formed the protoliths of high-grade metasediments of the Serre, those of the Aspromonte–Peloritani Unit and probably even those of Castagna unit, all these metasediments were intruded by Neoproterozoic–Cambrian magmas so they are older.
2. Panafrican orogenesis and consequent assemblage of blocks (having East and West gondwana affinities) involved these terranes as evidenced by metamorphism between 573 ± 3 Ma (this paper) and 535 Ma (Williams et al. 2012). In Neoproterozoic–Cambrian age (543–545 and 579 Ma), bimodal magmatism affected these terrains (Fig. 9).
3. Uplift and extensional tectonic in the Ordovician times with rifting and opening of Ordovician basins (Fig. 9; Acquafredda et al. 1991). This tectonothermal activity is documented in zircons of high-grade metasediments of the Serre, metabasites, metagabbros and augen gneisses interpreted as resetting ages clustering around 489, 442 and 453 Ma in all considered samples (Figs. 4b, 5a, 6b) (Schenk 1984, Trombetta et al. 2004; Micheletti et al. 2007). Evidences of deposition in Ordovician–Silurian times are present in detritic zircons of Mandatoriccio micaschists (451 Ma in Fig. 7a; Langone 2008) and in garnet–biotite gneisses (457–475 Ma Fig. 6a; Laurita et al. 2015) as well as in very low-grade metasediments of both Calabria and Sicily revealed by zircon ages of included porphyroids and meta-andesite (Trombetta et al. 2004; Martín-Algarra et al. 2014).
4. Variscan orogenesis involved all described rocks recording zircon ages in the range 370–270 Ma only in high-grade metamorphites (Fig. 6) in which the enough high temperatures and intense partial melting (Fornelli et al. 2002) caused the regrowth or recrystallization of zircons.

## Additional file

10.1186/s40064-016-1839-8 Analytica data of U-Pb zircon ages derived from literature.
